# How Willing Are Adolescents to Record Their Dietary Intake? The Mobile Food Record

**DOI:** 10.2196/mhealth.4087

**Published:** 2015-05-29

**Authors:** Carol Jo Boushey, Amelia J Harray, Deborah Anne Kerr, TusaRebecca E Schap, Stacey Paterson, Tanisha Aflague, Marc Bosch Ruiz, Ziad Ahmad, Edward J Delp

**Affiliations:** ^1^University of Hawaii Cancer CenterUniversity of HawaiiHonolulu, HIUnited States; ^2^School of Public HealthCurtin UniversityPerthAustralia; ^3^National Cancer Institute (NCI), National Institutes of Health (NIH)Cancer Prevention Fellowship Program, Division of Cancer Prevention, Division of Cancer Control and Population SciencesBethesda, MDUnited States; ^4^Department of Dietetics and Human NutritionUniversity of KentuckyLexington, KYUnited States; ^5^Qualcomm, Inc10160 Pacific Mesa Boulevard, Office 605kSan Diego, CAUnited States; ^6^Department of Electrical and Computer EngineeringPurdue UniversityWest Lafayette, INUnited States

**Keywords:** adolescents, children, dietary assessment, mobile food record, novel technology

## Abstract

**Background:**

Accurately assessing the diets of children and adolescents can be problematic. Use of technologies, such as mobile apps designed to capture food and beverages consumed at eating occasions with images taken using device-embedded cameras, may address many of the barriers to gathering accurate dietary intake data from adolescents.

**Objective:**

The objectives of this study were to assess the willingness of adolescents to take images of food and beverages at their eating occasions using a novel mobile food record (mFR) and to evaluate the usability of the user confirmation component of the mFR app, referred to as the “review process.”

**Methods:**

Mixed methods combining quantitative and qualitative protocols were used in this study. Adolescents (11-15-year olds) attending a summer camp were recruited to participate in the study. First, the participants were asked to take images of foods and beverages consumed as meals and snacks for 2 consecutive days using the mFR app running on an iPhone and the number of images taken was noted. This was followed by focus group sessions to evaluate usability, which was analyzed by content and themes. After using the mFR, a think-aloud method was used to evaluate the usability of the mFR method for reviewing system-identified foods (ie, the review process). A usability questionnaire was administered at the end of all activities.

**Results:**

The mFR was accepted by the majority of the 24 boys and 17 girls (n=41) but varied according to gender and eating occasion. Girls were significantly more likely than boys to capture images of their eating occasions (Fisher exact test, *P*=.03). Participants were more likely to take images of their breakfasts (90%, 36/40) and lunches (90%, 72/80) and least likely to capture afternoon and evening snacks, 54% (43/80) and 40% (32/80), respectively. The major themes from the focus groups with regard to using the mFR were games, rewards, and the need to know more about why they were using the app. Results of the usability questionnaire indicated that including a game component would be important to increase willingness to use the mFR, and a high majority of the participants indicated a willingness to use the mFR for 7 days or more. The image review process was found to be easy to use except for some confusion with overlapping markers on the screen.

**Conclusions:**

The adolescents’ experiences with and feedback about the mFR highlighted the importance of increased training, reminders, entertainment (eg, games), and training with practice in using the device to capture complete dietary intake as part of their active lifestyles.

## Introduction

Collecting information about dietary intake from children and adolescents is challenging. Developmental stages add to the complexity of deciding whether to gather information from the parent, the child, or both. Burrows and colleagues [1] performed a systematic review of validation studies that compared reported estimated energy intake from dietary assessment methods with the method of doubly labeled water as a biomarker for total energy intake among children aged 6 months to 21 years. The methods that provided the most accurate estimate of energy intake were the estimated dietary record as completed by the parent for 0.5-4-year olds, the multiple-pass 24-hour dietary recall as reported by the parent for 4-11-year olds, and the dietary history as reported by adolescents aged 16-21. However, a recommended method for 12-15-year olds did not emerge in their review. For early adolescents (11-14-year olds), the value of parental assistance remains equivocal. When girls without parental assistance completed dietary records at ages 10, 12, and 15, Bandini and colleagues [2] found that differences between estimated energy intakes compared with total energy expenditure calculated using the doubly labeled water method widened with increasing age. In a separate study, completion of dietary records with parental assistance was found to be associated with underreporting of energy intake by 20% and 33% at 11 and 12 years of age, respectively [3]. These latter results suggest that parental assistance for completion of dietary records among early adolescents does not enhance accuracy.

Livingstone et al [4] noted that assessing dietary intake in adolescents between 11 and 14 years of age is particularly problematic because the novelty of recording food wears off and the assistance from parents is no longer preferred. During focus group sessions [5], adolescents reported conventional dietary assessment methods, such as 24-hour dietary recalls, food frequency questionnaires, and dietary records, to be burdensome. Findings from the focus groups strengthen the need to explore more acceptable methods of assessing diet in adolescents in an effort to improve cooperation, which may lead to more accurate reports of dietary intake.

Taking images of eating occasions has been proposed as a method for reducing burden on adolescents participating in dietary studies [5]. When a group of 30 early adolescent boys and girls aged 10-14 years was asked to take photographs of their eating occasions for 1 day using disposable film cameras, all but 1 child complied; the majority took images for more than 1 day [5]. Of those taking images, 100% liked the method, compared with 35% liking the written dietary record and 52% liking the interviewer-administered 24-hour dietary recall. The children of this generation were born into a digital age, resulting in generational changes, such as difficulty sustaining attention to a specific task [6]. One barrier at the forefront of a mobile Internet world is that many children may attend a school that no longer teaches penmanship [7]. Digital technology, including social media, is shaping students’ writing and may lead to an increased use of informal spelling and truncated expression (eg, tweets). Thus, methods that rely on handwriting may present barriers. Use of technologies, such as Web- and mobile-based apps, may address some of the burdens and barriers to gathering accurate dietary data from children using recording methods they are familiar with, such as taking images. The rapid advancement of technology and mobile phones has motivated researchers to develop a mobile food record (mFR) to address barriers reported by adolescents [8].

**Figure 1:**
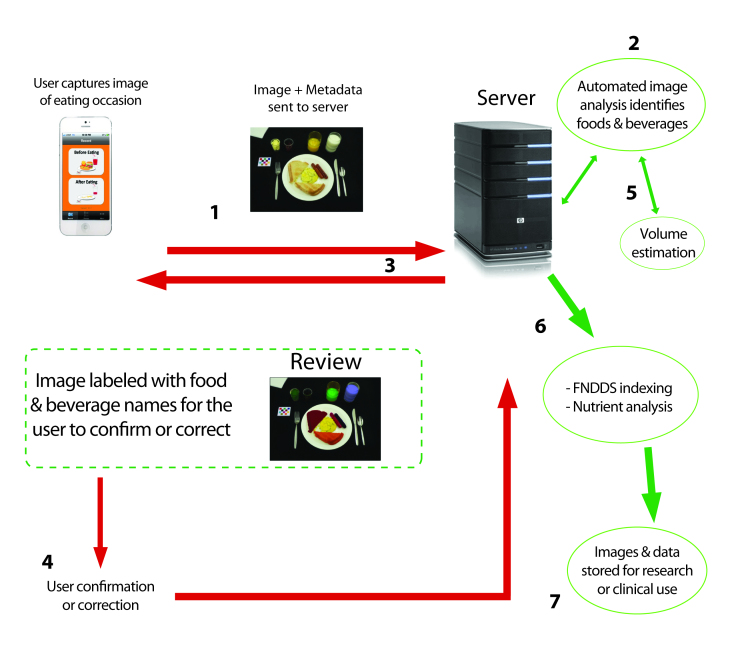
Diagram of the Technology Assisted Dietary Assessment (TADA) system that starts with capturing an image with the mobile food record (mFR).

The mFR allows users to take images of their food and beverages at eating occasions, which could address the barriers of hand-written pen and paper or digital entry methods that still require spelling and focused entry. Image analysis methods have the potential to automatically identify foods and beverages in the image and estimate volumes [9-12] (Figure 1). For the captured images to be useful for analysis, the user must capture all foods and beverages before and, if applicable, after consumption. A fiducial marker, which resembles a checker board square, is included in the images as a color and size reference to help with the reconstruction of a three-dimensional environment that allows for estimation of the volume of the foods and beverages [13-16]. In addition, as shown in Figure 1, the system includes a user confirmation component referred to as the “review process” [17]. This step is important as a predominantly automated system requires a method to identify new foods (whether new to the system or new to market), correct foods that are misclassified, or identify attributes that cannot be captured in an image (eg, no salt, artificially sweetened). Figure 1 shows each of the steps involved in capturing an image: (1) A user captures an image of an eating occasion that is sent to a server. (2) The image undergoes image analysis to identify the foods and beverages. (3) The labeled image is returned to the user for the “review process” as shown in the dotted line. (4) The user confirms the automatic labels or corrects the labels. (5) The image is returned to the server for final identification and volume estimation. (6) Identified foods and amounts are matched to the Food and Nutrient Database for Dietary Studies for nutrient analysis. (7) Images and data are stored on a server for use by researchers or clinicians. Schap et al [18] showed that adolescents can look at images of their eating occasions and identify the foods in the images up to 14.5 hours postprandially. Thus, the utility of using images in dietary assessment appears to have great promise.

The Pew Research Center recently reported that as of 2013, 78% of adolescents aged 12-17 years in the United States have their own mobile phone and nearly half of those (47%) own smart phones [19,20]. These reports emphasize that mobile phones have become “indispensable modes of teen communication.” The widespread penetration of mobile phones, particularly smart phones, with their increased computing power would suggest that these devices open up new opportunities for dietary data collection. They also have the potential to engage adolescents in the task. There is, however, a need to evaluate the usability and acceptability of mobile apps [21,22] and whether adolescents are willing to use a mobile phone to capture images of their eating occasions throughout the day. Therefore, the objectives of this study were to assess the willingness of younger adolescents to take images of food and beverages at eating occasions and whether willingness varied by gender, to evaluate the beta version of the review process component of the mFR and to assess acceptability and usability of the mFR.

## Methods

### Study Participants

Data were collected from adolescents participating in a residential summer camp on the Purdue University campus in 2010. There were 41 adolescents (17 girls and 24 boys) between 11 and 15 years of age who participated in this study. The active camp environment included participation in organized sports, hiking, river rafting, rock-wall climbing, relay races, swimming, bowling, social events, classes, free time, and attending a minor league baseball game. The activities described in this study were an add-on to the original objective of the residential camp, which was to establish dietary calcium requirements for bone health [23]. Participants received an incentive of US $100/3 weeks of the residential camp. No additional incentive was provided for participation in the add-on study reported here. The study methods described here were approved by the Purdue University Institutional Review Board, and informed consent and assent were obtained from the adolescents and their parents, respectively.

### Study Design

**Figure 2:**
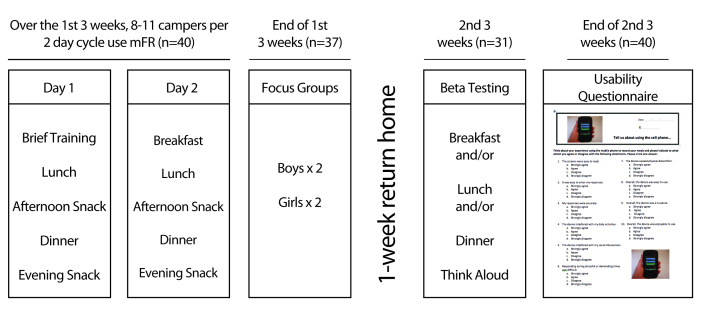
Mixed methods study design with quantitative and qualitative protocols.

This mixed-methods study sequentially used quantitative and qualitative data-collection methods and analyses to better explore adolescents’ willingness to use the mFR [22,24]. The camp was held for 3 weeks and included 2 sessions, with a single week at home between the sessions (Figure 2). An iPhone 3GS was distributed to each participant in 2-day cycles during the first session (3 weeks) of the camp. After breakfast on day 1 of the 2-day cycle, the participants were briefly introduced to iPhone 3GS and the mFR app Technology Assisted Dietary Assessment (TADA) installed on each mobile phone. Phone, data services, entertainment apps, and social media apps were preinstalled on each mobile phone. Internet and Internet-accessible apps were restricted to minors. They were instructed to take images of every eating occasion, including all meals, snacks, and beverages, with the fiducial marker. During the 2 days, the research staff did not remind the participants to capture images of their eating occasions. At the end of the first 3-week session of the camp, focus group sessions were conducted separately with boys and girls to obtain qualitative feedback about using the mFR.

**Figure 3:**
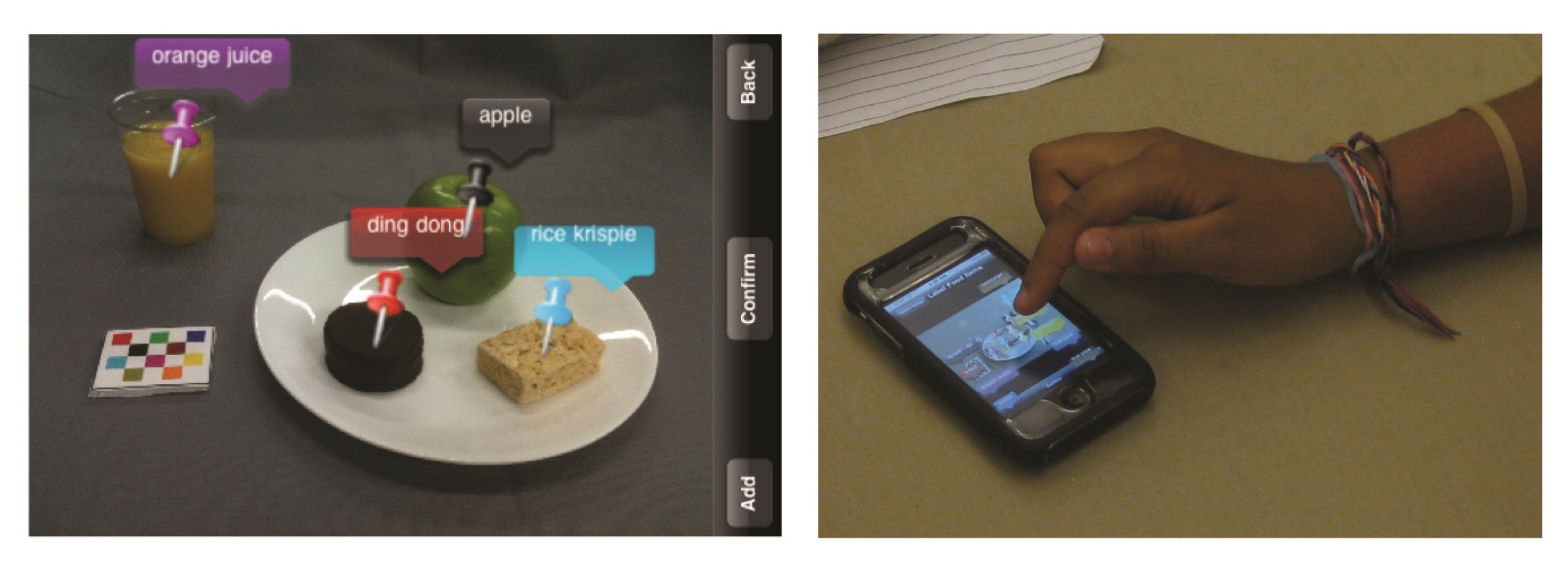
Beta testing the review component. The user selects an eating occasion to review. The before-eating image is in landscape view as displayed on the left. Foods are denoted with pins and the food label appears on a color coordinated bubble. To correct a misidentified food, the user taps the bubble associated with the food as shown on the right and selects the correct food from a list of foods (not shown).

During the second 3-week session of the camp, a subset of the same children used the iPhones again in groups of 8-11 at a time for breakfast and/or lunch and/or dinner. This data-collection protocol differed from the first session, in that the participants were monitored during the image capture process at each meal. At the end of the day, participants were provided instructions according to the think-aloud method [25], after which each participant used the beta version of the review process component on the mFR while a research staff observed (Figure 3). This process enabled the staff to follow the flow of task completion and take notes of the participants’ dialog. At the end of the second 3-week session, participants completed a usability questionnaire to solicit objective feedback about their experiences using the mFR.

### Evaluation of Willingness to Take Images

The camp situation was unique in that the eating occasions were limited to breakfast, lunch, an afternoon snack, dinner, and an evening snack. A 4-day cyclic menu consisting of the aforementioned eating occasions was used. Each camper was provided with adequate food to maintain his/her weight, according to his/her estimated energy requirements, which was monitored on a daily basis. No other foods or beverages were allowed. Over the 2 days of using the mFR, each participant was expected to capture a before- and an after-eating image (image pair) for each eating occasion. It was acceptable for participants to also only capture a before-eating image because they were expected to finish all foods and beverages provided as part of the main study protocol. Thus, a usable image was considered to be either an image pair or just the before-eating image. Usable images were considered indicative of participants’ willingness to comply with using the mFR. The set eating occasions allowed for the identification of the number of usable images and to classify participants as demonstrating high, moderate, or low willingness. One boy was unable to complete the task on both days due to reasons unrelated to this study and was excluded. For the remaining participants, the minimum expected total number of images was 360 (40 participants with 9 eating occasions, including 1 breakfast, 2 lunches, 2 dinners, 2 afternoon snacks, and 2 evening snacks). The boys and girls were classified as having taken 8-9 images (high willingness), 6-7 images (moderate willingness), and 3-5 images (poor willingness). At the end of each day, the research staff downloaded the mFR images captured using the TADA app to an Apple computer. The images were systematically reviewed by a trained analyst to enumerate only those containing images of the known meals or snacks.

### Focus Group Sessions

Focus groups were convened to explore the issues influencing participants’ willingness to capture images. The same script was used for all of the focus group sessions. All the girls attended 1 of the 2 girl-only focus group sessions. Three boys were unable to attend 1 of the 2 boy-only focus group sessions due to reasons unrelated to this study. Each focus group was led by 2 experienced moderators. Examples of questions relevant to this paper were as follows: “If we were to use these phones with other groups of teenagers, what could we do to help them remember that the main reason they have the phone is to take images of their meals and snacks?” Follow-up questions to this topic were “We have heard a lot of ideas about getting people to use the app, I just want to follow-up and see if anyone has more to add, what would motivate you to use the app each time you eat?” Another topic was “What did you like most about using the phones?” Copies of the entire script can be requested from the corresponding author of this paper. Two support staff hand-recorded each session and then pooled their notes. All staff present at a session reviewed the notes to achieve final consensus on the nature of the participants’ responses and any unscripted probes from the moderators. Two staff, other than the recorders, independently reviewed and analyzed the notes, categorizing them according to content and common themes. After consensus, these were finalized.

### Usability Testing a Beta Version of Image Review

Usability testing of a beta version of the user review process component of the app was used to identify potential improvements. Before the usability testing of the image review component, the children took images of at least 2 meals using the mFR. Thus, during the usability testing, all children were reviewing images of their own meals and were instructed to verbalize everything they were thinking as they decided which button to press first, and in every subsequent step, using the think-aloud method [25]. Participants were told that staff would not intervene or interrupt unless asked. Staff hand-recorded the key phrases verbalized by the participants. Standardized, nonthreatening prompts were used when a participant stopped verbalizing their thoughts before an interaction with the mFR. At the end of a session, the staff clarified their written notes with the participant. Two staff persons compiled the notes as related to the review process of the first image and subsequent image(s) and summarized common suggestions and common ease-of-use steps.

### Usability Questionnaire

A usability questionnaire was used to further inform behaviors related to using the mFR. The questionnaire was mainly composed of forced choice questions with 5 responses of “strongly disagree” to “strongly agree” and several open-ended questions [26,27]. Examples of questions relevant to this paper were related to the use of fiducial marker (eg, “I found the fiducial marker easy to use.”) and carrying 2 mobile phones. Open-ended questions included “How long would you record what you eat?” with response units of days, weeks, and months; and “What did you like the most about using the TADA iPhone?” Copies of the questionnaire can be requested from the corresponding author.

### Data Analysis

Willingness to take images was evaluated objectively by enumerating images captured of the known eating occasions. Each image was classified as being taken of a particular meal or snack. Information about each image was entered into a database, including whether the image was the before- or after-eating image and useful metadata (ie, date and time stamps). Data from the image evaluation and the questionnaire were entered into a Microsoft Access database (Microsoft, Redmond, WA, USA) and then imported into an SPSS version 17 database (SPSS Inc, Chicago, IL, USA) for data analysis. Statistical examination to compare girls and boys included frequencies, two-by-two tables, Chi-square test, Fisher exact test, and independent samples *t* test. Statistical significance was set at *P*<.05.

## Results

The classification of participants by ethnicity is shown in Table 1, and the characteristics of the boys and girls in this study are shown Table 2. During the first session, the participants used the mobile phones running the TADA app for 2 days and all mobile phones were returned with no damage. While in possession of the mobile phone, each participant had an opportunity to take images of 9 eating occasions. Of the 360 images expected as a minimum for the group of 40 participants, 246 (or 68.3%) of images were captured. There was no difference in the mean number of images captured by boys compared with girls (5.78 and 6.65 images, respectively). However, when examined by categories of high willingness (3 boys and 6 girls), moderate willingness (9 boys and 9 girls), or poor willingness (11 boys and 2 girls), girls were significantly more likely to capture images than boys (Fisher exact test *P*=.03). The participants were also more likely to take an image of the 1 breakfast (36/40, 90%) and the 2 lunches (72/80, 90%) than of the 2 dinners (63/80, 79%). Images of the afternoon and evening snacks were least likely to be captured at 54% (43/80) and 40% (32/80), respectively.

**Table 1:** Classification of study participants by ethnicity.

Ethnic group	Boys n (%)	Girls n (%)	Total^a^ N (%)
Hispanic	22 (91.7)	17 (100)	39 (95.1)
Non-Hispanic	2 (8.3)	0 (0)	2 (4.9)

^a^There were a total of 24 boys (59%) and 17 girls (41%) in this study (N=41).

**Table 2:** Characteristics of adolescents using the mobile phone food record (mFR) while attending a summer camp.

Characteristics	Boys		Girls	
	Mean	SD	Mean	SD
Age, years	13.9	0.9	13.5	1.0
Weight, kg	73.0	25	67.6	20
Height, cm	167.1	9.1	158.6	5.1
Body mass index	25.7	7.2	26.9	8.0

In the focus groups, participants were asked what they liked best about using the mobile phones; the theme mentioned most often were games and other forms of entertainment installed on the device (Textboxes 1 and 2). In response to the question, “What would motivate you to use the app each time you eat?” the major themes were positive feedback or recognition and some humor for the girls, and rewards and reminders for the boys (Textboxes 1 and 2). Suggestions for improvement included incentives or rewards for every image taken (eg, tokens, points), reminders (eg, a screensaver with the fiducial marker, friendly alarms, or texts), “cooler” colors, money, or something appearing on the screen after taking an image (eg, fireworks, “Awesome!”).

**Summaries of responses to some of the questions from the girls who participated in separate focus group sessions.**

1. What did you like most about using the phones?
  - Games
  - Facebook
  - Internet
  - It had a case
  - Calling
  - Tracking device that provides information if people steal it.
2. What would motivate you to use the app each time you eat?
  - Knowing that others are using/doing the same thing as you.
  - Reward for a week straight with adding fun apps if we take all our pictures. Even a reward after 2 straight days of using the app.
  - Different funny pictures pop-up every time we take a picture. Maybe even saying “good job” and laughing.
  - Provide entertainment with pop-up pictures.

**Summaries of responses to some of the questions from the boys who participated in separate focus group sessions.**

1. What did you like most about using the phones?
  - Games
  - YouTube
  - Music
  - Good for passing time
  - Apps
  - Did not get in trouble for using it
  - Keeping up with the World Cup
  - Internet
2. What would motivate you to use the app each time you eat?
  - Money
  - Extra money
  - A picture that gives an “awesome” after taking the image or “congratulations” with fireworks in the background.
  - A contest—whoever remembers to take the most photos of their meals wins something (eg, money).
  - If the phone had a bleep or vibrate around meal time.
  - Something pops up on the screen around meal time to remind you
  - Receiving a text message from TADA.
  - Alarms

The usability findings of the review process were useful (Figure 3). The most common reaction with regard to the review process was “confusing because there are too many pins overlapping.” Some individuals wanted to tap the pin rather than the bubble containing the name to confirm or relabel the food. Many of the participants did not know what to do until the process was explained; however, most were able to figure out the process without instruction. The majority described the second time as “easier,” and once the process was successfully mastered, many were observed as going so fast as they unintentionally deleted some pins.

The usability findings from the questionnaire pertaining to the app as a whole are shown in Table 3. With regard to using the fiducial marker, the majority of the adolescents (32/41, 78%) agreed that the fiducial marker was easy to use, carry around, and include in the images of their eating occasions. Some of the participants had their own mobile phones, and therefore, carrying the iPhone was an additional mobile phone to carry. However, there was a strong agreement that carrying 2 mobile phones was easy. Nearly all of the boys and girls agreed that understanding the purpose of the TADA app running on the iPhone would have been helpful. When asked “How long would you be willing to record what you eat using the TADA iPhone app?,” 39% (16/41) wrote a value between 45 and 90 days, 32% (13/41) wrote 30 days, 10% (4/41) wrote a value between 10 and 15 days, 12% (5/41) wrote 7 days, and 7% (3/41) wrote 0-1 day. The most common response (30 of 40 responses) to the question “List what you liked most about using the TADA iPhone” was entertainment oriented (eg, games, Internet, taking pictures). Among those individuals classified as demonstrating high willingness to take images, all listed “games” in response to this question.

**Table 3:** Responses to statements in usability questionnaire among adolescents after using the Technology Assisted Dietary Assessment iPhone (n=40).

Statement, as presented		Agreed by boys^a^ n (%)	Agreed by girls^b^ n (%)
**I found the fiducial marker easy to**			
	Use	21 (91)	11 (65)
	Carry around	20 (87)	11 (65)
	Include in the picture of my meals	20 (87)	12 (71)
Understanding the purpose of the TADA iPhone app would have been helpful		20 (87)	17 (100)
Carrying 2 telephones is easy (n=31^c^)		16 (84)	12 (100)

^a^Boys (n) = 23

^b^Girls (n) = 17

^c^Refers to carrying TADA iPhone and the personal mobile phone. The numbers of boys and girls who were carrying both were 19 and 12, respectively.

## Discussion

### Principal Findings

This study assessed adolescents’ willingness to take images of eating occasions using a mobile phone and observed their interactions with completing the beta version of the review process component of the app. A notable strength of this study was an objective marker for eating occasions. Just under half of the participants were moderately willing to capture images of their eating occasions indicating that some type of reminder system would be important for capturing total diet record. Girls appeared to be more willing to take images than boys, suggesting that more effort is needed to make this type of activity more compelling to boys. During the focus group sessions, the participants volunteered the importance of incentives and reminders to assist them in remembering to take images of every eating occasion. Whereas only basic instruction was provided about how to take the images, more extensive training may clarify the importance of taking images and might enhance willingness and cooperation [26]. Once the participants mastered the steps of the review process component, the majority were observed to quickly tap the labels on the screen much like approaching a game. The complaint of the overlapping pins encountered during the beta testing of the review process was addressed by adding a feature to enlarge the screen. To prevent accidental removal of a pin, a confirmation step was added.

Adolescents especially liked the game apps loaded on the iPhone. All of the individuals objectively classified as demonstrating high willingness also wrote “games” on the questionnaire as the item they liked most about the telephone. This would suggest that games may enhance the level of interaction with the telephone, which then transfers to remembering to take images of foods and beverages at eating occasions. Providing a mobile phone with preloaded games and apps or offering a selection of games or apps may provide additional motivation for cooperation. A study by Baranowski and colleagues [28] suggests that games themselves are a promising feature for enhancing behavior change in children. Overall, these results would support modifying the TADA app to incorporate aspects of gaming, which in turn may benefit usability and cooperation.

During the focus group sessions, both boys and girls expressed a desire to know why taking images of what they eat would be useful or important. Based on the questionnaire responses, nearly all participants agreed that understanding the purpose of the mFR would be helpful. Thus, full transparency would be important in gaining commitment and cooperation from adolescents. In this study, the amount of information initially shared with the participants was limited to determine salient issues. Thus, even with modern technology, the need for full disclosure remains important to adolescents.

Adherence was most influenced by the type of eating occasion (eg, snacks being the worst). Compliance with the dinner meals was substantially lower than the breakfast and lunches. The lower compliance with the dinner meals can partially be explained by the 1-dinner meal that was a picnic on the grass during a minor league baseball game. This particular meal was the most noncompliant meal and reinforces the importance of incorporating reminders to engage an individual when such occasions occur. The minimal training provided in this study was likely inadequate and highlights the need to develop appealing training to aid in the aforementioned situations [26].

Although carrying and using the fiducial marker and carrying 2 telephones intuitively seem burdensome, the majority of the participants agreed that dealing with the fiducial maker and 2 mobile phones was easy. Boys appeared to be more positive than girls with regard to using and carrying the fiducial marker. By contrast, the girls were more likely to state that the protective covers on the telephones were attractive, and if they also included space for the fiducial marker this may be additionally useful. Such a feature could promote a more positive preference among girls regarding the use of a fiducial marker. However, to improve the number of images including a fiducial marker, participants suggested incorporating an automated system into the app to alert the user when the fiducial marker was not visible. In response to this, the opening screen of mFR TADA app has been modified to include a cartoon of the fiducial marker as a reminder [29]. In addition, the app has been modified to detect the presence of the fiducial marker and improve clarity of the image [16,30]. Further, the user settings were modified to allow users to select favorite background colors, a suggestion raised in the focus group sessions.

### Limitations

A limitation of this study was the hand recording of the think-aloud method, as well as questions and discussions during the focus group sessions being interpreted through the understanding of the recorders and not amenable to independent reanalysis by other coders. Another limitation was the time of year. The summer is an active period for adolescents and this could influence their ability to remember to take images of their eating occasions. During the school year, there may be other barriers, such as restriction of mobile devices in the school environment. By contrast, daily activities during the school year may be more routine than in an active camp environment. Nonetheless, an exploration of challenges with the community dwelling situation and the school environment is in order. Some additional next steps would include comparing results with biomarkers and determining the lowest age at which children are able to capture images either by themselves or with the aid of parents or other adults. Surrogate parent and care-provider reporting was found to be promising with few limitations [31]. Nonetheless, allowing young children to capture images of their eating occasions may ameliorate identified shortcomings.

### Conclusions

The mFR was accepted by the majority of adolescents in the study, but varied according to gender and eating occasion. The adolescents’ experience of using this novel method of technology to assess diet and their feedback highlighted the importance of reminders, games, and more training and practice in using the TADA app. Addressing these recommendations may assist adolescents with measuring their dietary intake as part of their active lifestyles.

## Abbreviations

mFR: mobile food record
TADA: Technology Assisted Dietary Assessment
